# Cutaneous B-cell lymphoid hyperplasia successfully treated with oral abrocitinib: A case report

**DOI:** 10.1016/j.jdcr.2026.09.019

**Published:** 2026-09-20

**Authors:** Kennedy Sparling, Daniella M. Allevato, David Glembocki, Christina Harview

**Affiliations:** a University of Arizona, College of Medicine, Phoenix, Arizona; b HonorHealth, Dermatology Residency, Scottsdale, Arizona; c US Dermatology Partners, Phoenix, Arizona; d Medical Dermatology Specialists, Phoenix, Arizona

**Keywords:** abrocitinib, cutaneous B-cell lymphoid hyperplasia, JAK inhibitor, refractory pruritic dermatosis

## Introduction

Cutaneous B-cell lymphoid hyperplasia (CBCLH) is an uncommon cutaneous lymphoproliferative disorder characterized by a broad spectrum of clinical presentations.^1^ Although approximately 30% of cases are idiopathic, CBCLH often arises secondary to identifiable external or internal stimuli, including infections, medications, trauma, neoplasms, and foreign substances.^2^ Accordingly, management is tailored to the underlying etiology and may include antibiotics, medication cessation, corticosteroids, immunomodulators, radiotherapy, and surgical interventions.^3^^,^^4^ In cases refractory to conventional therapies, escalation to rituximab (monoclonal anti-CD20 antibody), methotrexate, thalidomide, and topical imiquimod, have been reported.^5^, ^6^, ^7^, ^8^ However, the use of targeted immunomodulators, including Janus kinase (JAK) inhibitors such as abrocitinib, has not previously been reported to our knowledge. We report a unique case of refractory, disseminated, nodular CBCLH successfully treated with the JAK inhibitor abrocitinib, highlighting its potential as a novel therapeutic option for difficult-to-manage disease.

## Case report

A 78-year-old healthy female presented to the dermatology clinic with chronic, intensely pruritic nodules distributed on the upper and lower extremities. The nodules reportedly first appeared 24 years prior to presentation, temporally associated with a hysterectomy. Since the onset, the nodules have progressively increased in number, but have never involved the head/neck, genital region, or palms/soles. The patient identified severe pruritus as the most debilitating symptom, which she rated as 10/10, resulting in poor sleep since disease onset.

Physical examination revealed numerous flesh-colored, firm, dome-shaped nodules distributed across her upper and lower extremities (Fig 1, *A-C*). Prior allergy testing on 2 occasions was negative. Over the 24-year disease course, the patient had undergone multiple treatment options (oral doxycycline, narrowband UVB phototherapy, topical therapies (tacrolimus, clobetasol, and triamcinolone), and intralesional corticosteroid injections) without any significant improvement in pruritus.

**Fig 1:**
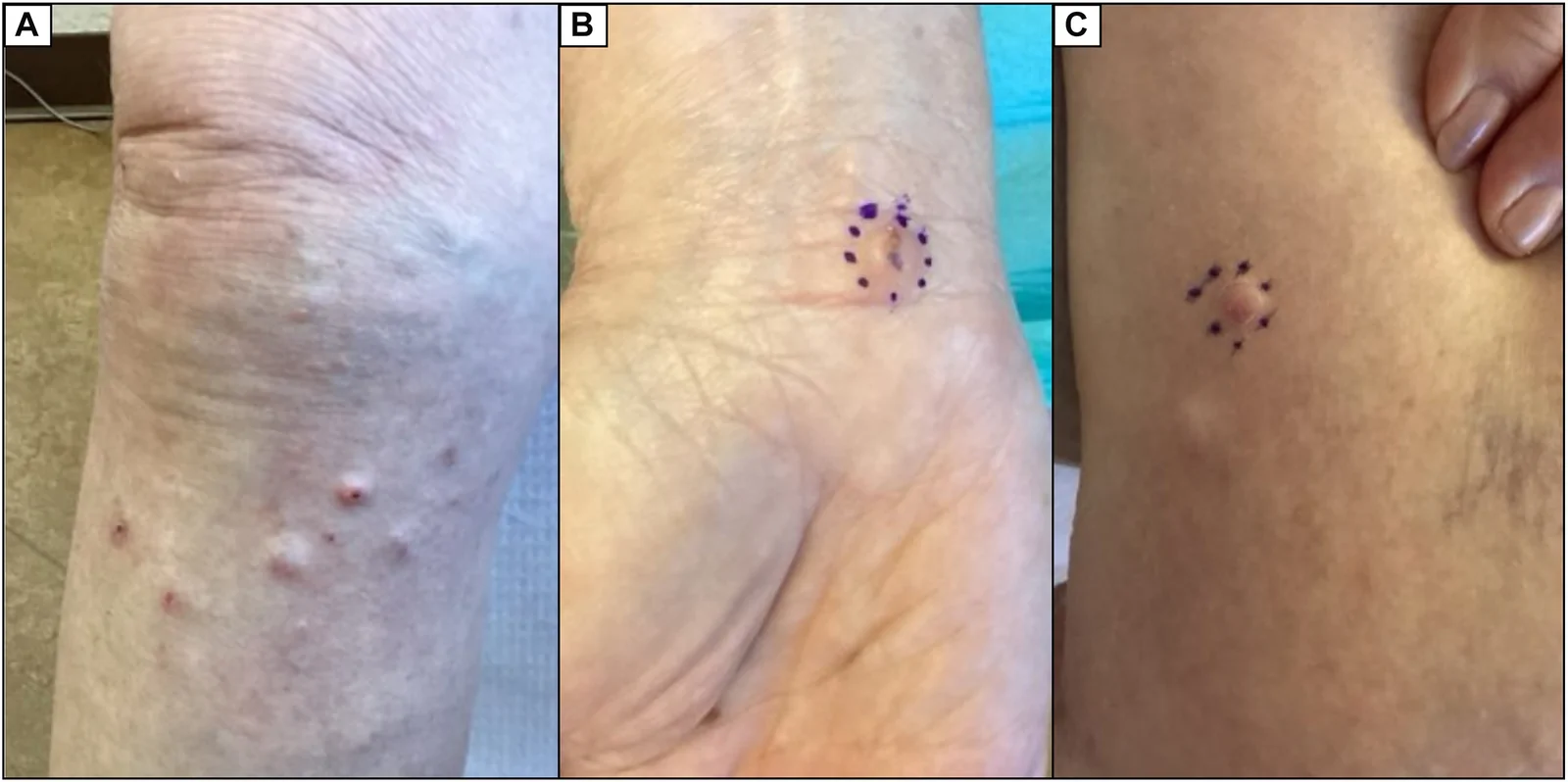
Clinical photographs of the lesions involving the right inferior knee **(A)**, ventral wrist **(B)**, and lateral thigh **(C)**.

Numerous skin biopsies had been performed, all of which were nondiagnostic. The most recent biopsy, in 2019, demonstrated epidermal acanthosis with hyperkeratosis, parakeratosis, and a dermal lymphohistiocytic infiltrate, leading to a working diagnosis of angiolymphoid hyperplasia by an outside physician. This was not concordant with the patient’s current physical examination; therefore, biopsies were obtained from 3 sites and evaluated using hematoxylin and eosin staining and immunohistochemical analysis. Histopathologic examination demonstrated marked chronic changes, including features resembling prurigo nodularis with dermal fibrosis and epidermal acanthosis (Fig 2, *A* and *B*). Numerous eosinophils were present, along with associated lymphoid hyperplasia. Immunohistochemical revealed a sparse CD3+ T-cell population, with the lymphoid infiltrate predominately composed of CD20+ B cells with germinal center formation (Fig 3). Prominent CD20+ B-cell proliferation has been described in chronic, reactive lymphoid infiltrates, particularly in longstanding lesions, and was felt to reflect disease chronicity in this case.

**Fig 2:**
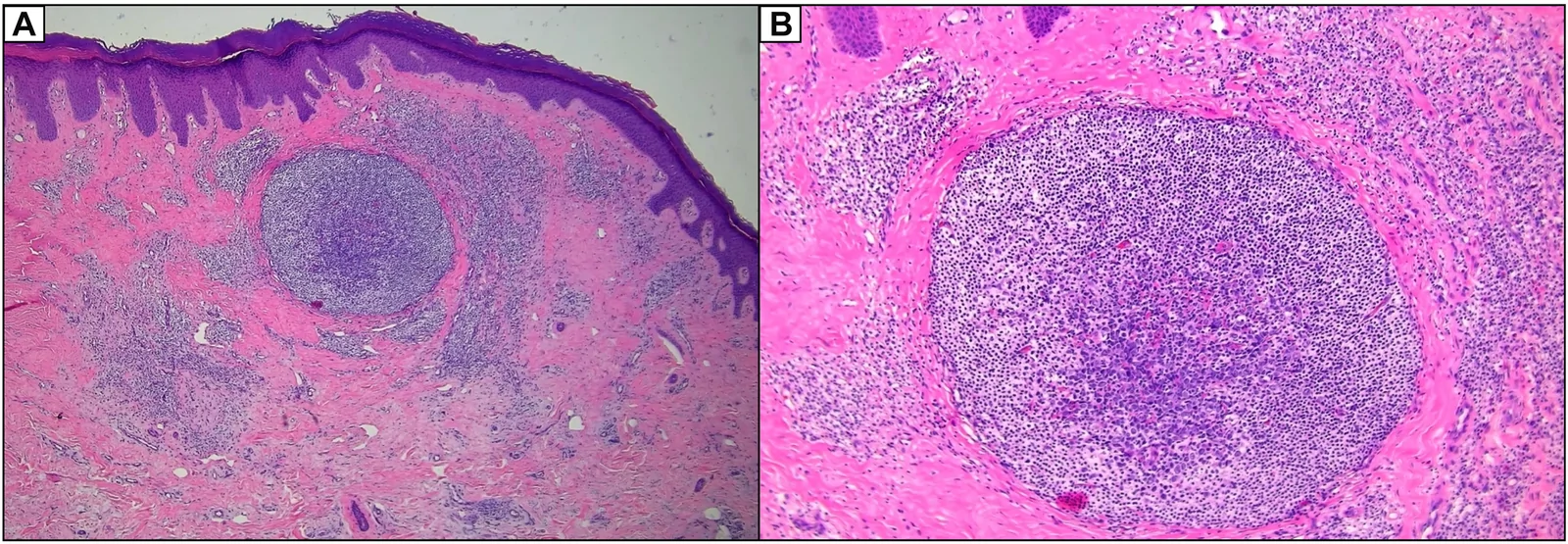
Hematoxylin and eosin (H&E) stained sections demonstrating epidermal acanthosis, dermal fibrosis and a dense inflammatory infiltrate with numerous eosinophils and lymphoid hyperplasia **(A)** 40× **(B)** 100×.

**Fig 3:**
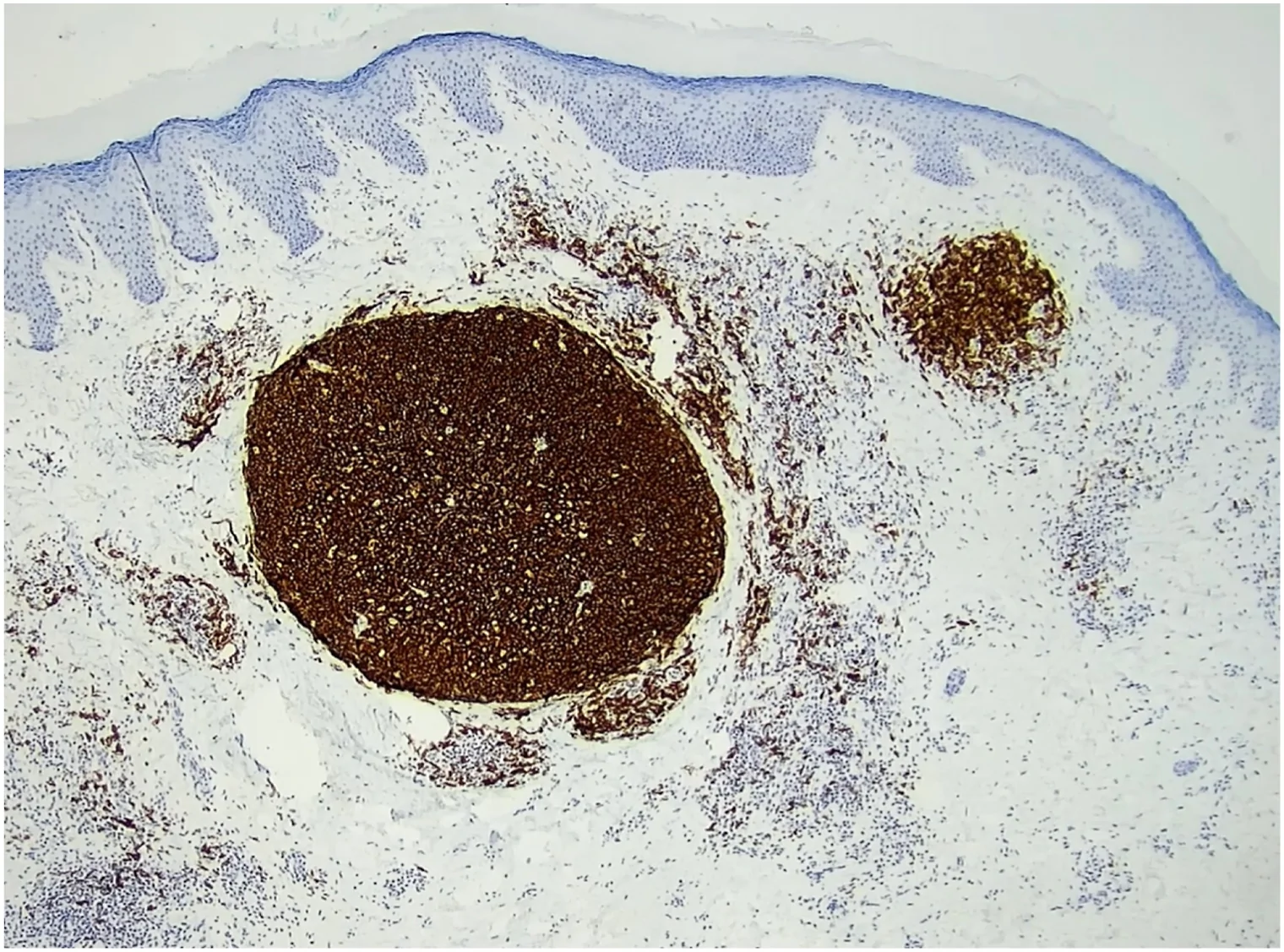
Immunohistochemical studies demonstrate a predominately CD20+ B-cell infiltrate with germinal center formation.

Laboratory evaluation demonstrated a comprehensive metabolic panel and complete blood count within normal limits. The lipid panel noted mild hypercholesterolemia with elevated low-density lipoprotein. Infectious disease screening was negative for QuantiFERON-TB Gold, coccidiomycosis serologies, hepatitis B and C, and human immunodeficiency virus. Lyme serology was not obtained, given the >20-year disease duration and absence of systemic symptoms or tick exposure.

Based on the clinical presentation and histopathologic findings, a diagnosis of CBCLH was established. Traditional systemic therapies were unsuitable due to current alcohol consumption (methotrexate) and medication interactions (mycophenolate mofetil, cyclosporine, acitretin, and azathioprine). After extensive counseling on the risks, benefits, and treatment options, oral abrocitinib was initiated at a dose of 100 mg daily. Within several days of initiation, she reported complete resolution of pruritus, with concomitant improvement in sleep quality. At follow-up several months later, nodule thickness and size were beginning to improve, and her pruritus score remained 0/10 (Fig 4). No adverse effects were reported during treatment. Notably, a 10-day interruption in therapy due to insurance-related issues resulted in recurrence, suggesting that continued therapy may be required.

**Fig 4:**
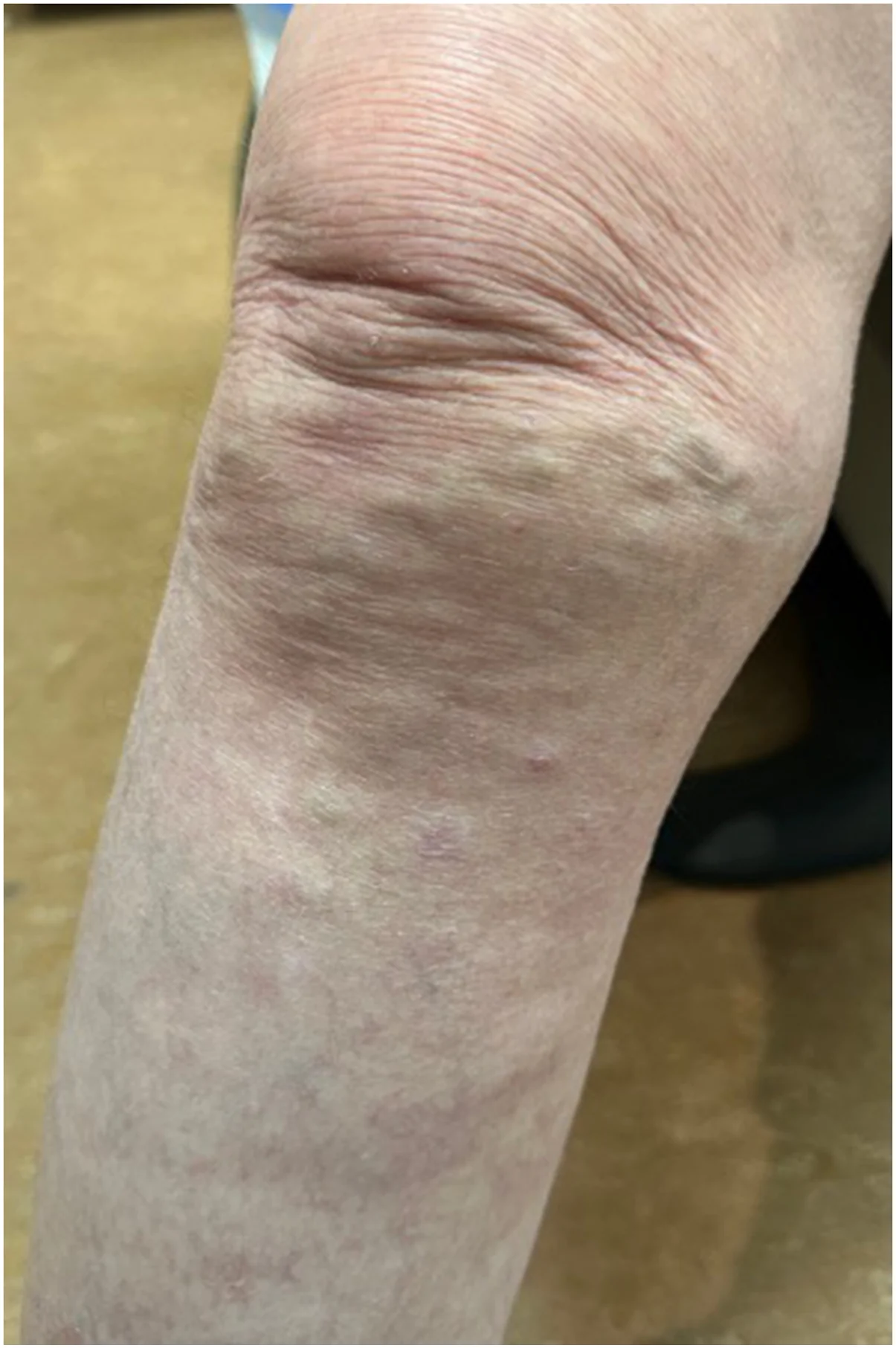
Clinical photograph of the improved lesions involving the right inferior knee.

## Discussion

CBCLH is a benign and uncommon cutaneous lymphoproliferative disorder characterized by the reactive proliferation of B lymphocytes within the dermis and superficial subcutis.^9^ The clinical presentation of CBCLH can be highly variable and encompasses solitary, grouped, or disseminated papules, plaques or nodules that range in color from red to violaceous.^10^ Our patient demonstrated the disseminated nodular subtype, which accounts for approximately 10% to 15% of CBCLH cases.^11^ Due to its diverse presentation and clinicopathologic overlap with other dermatologic entities, including cutaneous lymphomas and granulomatous disorders, the diagnosis is challenging and necessitates multiple biopsies.^10^^,^^12^ prurigo nodularis-like changes may accompany reactive lymphoid proliferations, as previously reported in a case of refractory prurigo nodularis with histopathologic features of angiolymphoid hyperplasia with eosinophilia.^13^

The pathogenesis of CBCLH is poorly understood and is thought to represent a reactive hyperplastic response to diverse antigenic stimuli. Reported triggers include infectious agents, *Borrelia burgdorferi*, as well as trauma, arthropod bites, and exposures to foreign materials, such as tattoos and hair dyes.^2^^,^^10^ Drug-induced cases and postvaccination reactions have also been reported.^2^^,^^14^^,^^15^ Approximately 30% of cases are considered idiopathic, with no discernible inciting factor.^2^ This etiologic heterogeneity underscores the importance of comprehensive diagnostic evaluation and individualized treatment regimens.

Therapeutic strategies for CBCLH are generally directed toward the underlying cause. *B* *burgdorferi* infection responds to oral antibiotic therapy,^16^^,^^17^ whereas drug-induced resolves following drug discontinuation. When idiopathic, management is challenging due to the lack of a defined target. Therapeutic options for localized lesions may include observation, topical or intralesional corticosteroids, topical tacrolimus, or surgical excision.^18^ In cases of extensive disease, the literature suggests systemic corticosteroids, intralesional or systemic interferon-alpha, or oral hydroxychloroquine, although supporting data are limited.^3^^,^^11^ In refractory CBCLH, prior case reports have noted successful responses to rituximab, methotrexate, topical imiquimod, thalidomide, and localized radiotherapy.^4^^,^^6^, ^7^, ^8^ To date, the use of abrocitinib, a selective Janus kinase (JAK) inhibitor, has not been reported for refractory CBCLH.

In this case, treatment with oral abrocitinib resulted in marked symptomatic improvement, highlighting its potential as a promising therapeutic option for managing CBCLH. By inhibiting JAK-mediated signaling pathways downstream of multiple proinflammatory cytokines, abrocitinib may modulate immune signaling contributing to B-cell–predominant inflammation, thereby addressing the underlying immune dysregulation characteristic of CBCLH.^1^^,^^19^ Moreover, JAK1 inhibition reduces pruritus through blockade of cytokine signaling pathways, particularly the interleukin-31 pathway, which is implicated in chronic pruritus.^20^^,^^21^ Longitudinal follow-up is warranted to evaluate the long-term response and safety.

CBCLH poses a diagnostic and therapeutic challenge due to its diverse clinical manifestations and complex etiology. The favorable response to abrocitinib in this case supports further investigation of JAK inhibition as a potential therapeutic strategy for refractory or idiopathic CBCLH. Improved understanding of disease mechanisms and treatment response may facilitate earlier diagnosis, more targeted management, and improved patient outcomes.

### Declaration of generative AI and AI-assisted technologies in the writing process

None.

## Conflicts of interest

None disclosed.
